# High frequency of mutations in *gyrA* gene associated with quinolones resistance in uropathogenic *Escherichia* *coli* isolates from the north of Iran

**DOI:** 10.22038/ijbms.2018.31285.7539

**Published:** 2018-12

**Authors:** Mohammad Shenagari, Masoud Bakhtiari, Ali Mojtahedi, Zahra Atrkar Roushan

**Affiliations:** 1 Department of Microbiology, Faculty of Medicine, Guilan University of Medical Sciences, Rasht, Iran; 2 Department of Biostatistics, Faculty of Medicine, Guilan University of Medical Sciences, Rasht- Iran

**Keywords:** Uropathogenic Escherichia coli, Quinolone, Antibiotic resistance, gyrA, PCR-RFLP

## Abstract

**Objective(s):**

Regarding the global burden of uropathogenic *Escherichia coli* (UPEC) infections, prevention and treatment of such infections play a significant role in healthcare management. The inordinate use of fluoroquinolones led to a worldwide spread of quinolone-resistant strains. Therefore, this study aimed to investigate mutations in codons 83 and 106 of *gyrA* gene in UPEC isolates in the north of Iran.

**Materials and Methods:**

This cross-sectional study performed on a total of 223 UPEC isolates which were recovered within 6 months in 2017. Isolates were identified and confirmed by standard microbiologic tests, and antimicrobial susceptibility testing was carried out by disk diffusion and E-test methods. PCR reaction was performed to amplify *gyrA* gene, and PCR-RFLP was performed using *BsiEI* and* BstUI *restriction enzymes to investigate mutations in *gyrA* gene.

**Results:**

The nalidixic acid, ciprofloxacin, ofloxacin, and norfloxacin resistance rates were 61.9%, 50.2%, 48.25, and 45.3%, respectively. Overall, 55.2% of *E. coli* isolates had a mutation in *gyrA* gene in codon 83, and 20.2% in codon 106. Also, 15.2% of isolates had simultaneously mutation. Moreover, a significant association was found between mutations in *gyrA* gene and quinolone and fluoroquinolones resistance pattern of UPEC isolates.

**Conclusion:**

Our results revealed a high level of quinolone resistance associated with the mutations in *gyrA* among the clinical isolates of UPEC in our region. To the best of our knowledge, this study is the first investigation on the role of *gyrA* alteration in quinolone resistance among UPEC isolates from the north of Iran.

## Introduction

Quinolones are one of the synthetic antibiotics which extensively used worldwide (1, 2). Urinary tract infections (UTIs) were treated by first-generation (acidic) quinolones, including nalidixic acid (1). However, the range of effectiveness has improved by alteration of the following generations. One of these changes was the addition of a fluorine atom at position C-6 of antibiotic molecules which leads to extensive powerful activity against different Gram-negative bacteria (3, 4). Fluoroquinolones bind to and impede the activity of topoisomerase II (DNA gyrase) and topoisomerase IV (*parC* and *parE*) (4). DNA gyrase comprised of two subunits A and two subunits B, which are encoded by the *gyrA* and *gyrB* genes, respectively (5).

Extensive and inordinate consumption of antibiotics over the recent years has been leading to increasing trends of antibiotic resistant bacteria (6, 7). Nowdays, with the advancement of antibiotic resistance mechanisms, the issue of antibiotic resistance become an important concern in the health systems (6, 8, 9).

In *Escherichia* *coli*, resistance to quinolones frequently occurs through mutation in *gyrA* and less often by *gyrB* genes, which catalyzes ATP-dependent DNA supercoiling (5). Some other mechanisms of *E. coli *resistance to quinolones and fluoroquinolones are through efflux pumps and reduced drug accumulation in the bacteria due to changes in the purine protein (4, 10). Many studies have revealed that mutations in a small parts of *gyrA* N-terminal (Amino acids 67 (Ala-67) to 106 (Gln-106)) leads to quinolones and fluoroquinolones resistance which is named quinolone resistance-determining region (QRDR) (11). Meanwhile, the most of the mutations arose in nucleotide 248 and 620, causes amino acids aspartic acid 83 and serine 87 alterations (12).

Among the point mutations, the most relevant altration is that on nucleotide 247 (Ser-83) of the *gyrA* gene (10, 13). In clinical isolates, the second most commonly observed mutation is at codon 87 of *gyrA* gene (14). Some studies have reported that resistant bacteria to quinolones had no mutation in the codon 83 *gyrA* gene. Also in some cases despite mutation in codon 83 *gyrA* gene, the bacteria were susceptible to quinolones. It has been supposed that such resistant strains may have a point mutation in other sites or along with codon 83 in the *gyrA* gene which may lead to high-level resistance to quinolones (5, 14). A few studies have surveyed the effect of the mutation on codon 106 in conferring resistance to quinolones (15). To our best knowledge, there is no report about the investigation on mutation in the codon 106 among clinical *E. coli* in Iran. 

Mutations in *gyrA* gene can be identified with several methods such as sequencing, single-strand conformational polymorphism (SSCP) and mismatch amplification mutation assay PCR (MAMA-PCR), but these techniques are quite expensive and time-consuming (16). PCR-RFLP is one of the best methods for identifying the point mutation in a sequence of DNA. Therefore, this study aimed to investigate mutations in codons 83 and 106 of *gyrA* gene in susceptible and resistant isolates of uropathogenic* E. coli* (UPEC) in the north of Iran.

## Materials and Methods

***Study design and bacterial isolation***

This cross-sectional study was performed to assess the importance of *gyrA* gene mutations in quinolone and fluoroquinolone resistance in 1250 urine samples (mid-stream, clean catch) which were collected in a period of 6 months from February 2017 to June 2017. Samples obtained from the patients with UTIs who referred to a tertiary hospital (Razi hospital) in the Rasht, the north of Iran. This study was in accordance with the declaration of Helsinki and approved by the regional Ethics Committee. The specimens plated on Blood agar (Quelab, Canada) and Eosin Methylene Blue agar (Pronadisa, Italy) plates. The plates were incubated overnight at 37ºC. Then, colonies with green metallic sheen were supposed as *E. coli* and identified by standard microbiological tests including Gram stain, oxidase, and differential biochemical tests including Triple Sugar Iron agar, Simmons' citrate agar, Christensen’s urea agar, Indole test, Methyl red and Voges-Proskauer tests. The confirmed isolates were stored in Trypticase Soy Broth (TSB) with 15% glycerol at -70ºC for long preservation.

***Antimicrobial Susceptibility testing***

All of the *E. coli *isolates were tested for susceptibility to quinolone and fluoroquinolone including nalidixic acid (30 µg), ciprofloxacin (5 µg), ofloxacin (5 µg), and norfloxacin (10 µg) (MAST, UK) by standard disk diffusion method on Mueller-Hinton agar medium (Merck, Germany) as described by the Clinical and Laboratory Standards Institute (CLSI) guidelines (17). Minimum inhibitory concentrations (MICs) were determined by E-test (Lioflichem, Italy) as described by the CLSI recommendation.* E. coli* ATCC 25922 strain was used as quality control purposes.

***DNA extraction and polymerase chain reaction (PCR)***

Genomic DNA of all isolates was extracted using High pure DNA template preparation kit (Roche, Germany) as stated by manufacturer instruction. PCR reaction was performed to amplify *gyrA* gene in Quinolone resistant determining region (QRDR) using specific primers, *gyrA*-F: 5’-GCT GCC AGA TGT CCG AGA T-3’, *gyrA*-R: 5’-TCC GTG CCG TCA TAG TTA TCA-3’. Reaction condition was initiated by pre-denaturation at 95 ºC for 5 min followed by 45 cycles (94ºC for 1 min 60ºC for 30 sec 72ºC for 30 sec) and final extension cycle (72ºC for 5 min). A 360 bp band on agarose gel containing DNA safe stain (Cinnagen, Iran) was visualized under UV Tran illuminator.

***PCR-Restriction fragment length polymorphism (RFLP)***

In order to detection of mutation in 83 and 106 codons of *gyrA* gene, PCR-RFLP was performed using *BsiEI* and* BstUI *restriction enzymes (Thermo fisher scientific Inc., USA). The source of *BstUI* restriction enzyme is *Bacillus stearothermophilus* U458 and, *BsiEI* was getting from an *E. coli *strain that carries the cloned *BsiEI* gene from *Bacillus* sp. The cutting site for *BstUI* is (5 CG CG 3 or 3 GC GC 5) and for *BsiEI* is (5…CGRY CG…3 or 3…GC YRGC…5). The 360 bp PCR products were digested using both enzymes simultaneously according to manufacturer guideline. The digested fragments were subjected to electrophoresis on a 2% agarose gel stained with DNA safe stain and was visualized under UV Tran illuminator.

***Statistical analysis***

Resistance rates among the isolates and comparisons of fluoroquinolones resistance and mutation in the *gyrA* gene were analyzed with Chi-square or Fisher's exact tests. A difference was considered statistically significant if the *P*-value was less than 0.05.

## Results

Among 223 isolated UPEC isolates, ciprofloxacin, ofloxacin, and norfloxacin resistance rates were approximately similar (50.2%, 48.25, and 45.3%, respectively), whereas a higher level of resistance to nalidixic acid was seen (61.9%) (Table1). Moreover, the full results of antibiotic susceptibility testing including MIC50 and MIC90 (MIC at which 50% and 90% of isolates were inhibited) of the tested isolates are shown in Table 2. 

In the present study, 55.2% of *E. coli* isolates had a mutation in *gyrA* gene in codon 83. Mutation in codon 106 occurred in 20.2% of cases. Also, simultaneously mutation (codons 83 and 106) was observed in 15.2% of isolates. 

Among wild-type strains there is one restriction site for *Bst*UI at nucleotide 42 which yields 42 and 318 bp fragments after digestion, while in mutants, *gyrA *gene attains another restriction site at nucleotide 149 (Serine → Alanine). So, three fragments (42, 107 and 211 bp) are observed after digestion and electrophoresis. Also, *Bsi*EI has two restriction sites at nucleotide 168 and 330 in wild-type strains which produces three fragments (390, 168 and 132 bp). If a mutation occurs in *gyrA* gene, a new restriction site developed at nucleotide 217, which produces 4 fragments (30, 168, 138 and 49).

The association between a mutation in codon 83, 106 or both and ciprofloxacin resistance pattern among *E. coli* isolates showed that mutation in codon 106 had the most effect on resistance to ciprofloxacin (Table 3). Our results revealed that simultaneously mutation in codon 83 and 106 conferred more resistance rate to norfloxacin among *E. coli* isolates (Table 4). In the present study, a mutation in codon 106 *gyrA* gene had the most effect on resistance to ofloxacin in *E. coli* isolates as such as ciprofloxacin (Table 5). Finally, *E. coli* isolates which had a mutation in both codons 83 and 106 *gyrA* gene were more resistant to nalidixic acid which was similar to norfloxacin (Table 6).

## Discussion

Regarding the global burden of UPEC infections, prevention and treatment of such infections play a significant role in healthcare management (18). Fluoroquinolones are an important class of antibiotics for the treatment of UPEC; however, the inordinate use of these agents led to a worldwide spread of quinolone-resistant strains, particularly in developing countries (19). In the present study, a remarkable rate of quinolones resistance (ranging from 45.3% to 61.9%) in 223 tested UPEC isolates were found. Despite the great discrepancy, the prevalence of quinolone-resistant UPEC isolates in our findings was consistent with the median values (range 14% to 71%) reported in different regions of the country (20-26). Based on the literature, the level of resistance to quinolones is increasing in other parts of the world, as well. Several reposts from Asian, European, African, and south-American countries indicate to the high prevalence of fluoroquinolones resistance even more than 50% and raised serious concerns (27-33). Meanwhile, our findings regarding the MICs of fluoroquinolones showed that the MIC ranges in resistant-strains is significantly high, and it seems that in clinical setting overcoming to this level of resistance even by using the higher dosages of MIC50/MIC90 values can be difficult. One explanation for such an observation in our findings may be due to the indiscriminate use of antibiotics in clinical settings, for example empirical therapy of uncomplicated UTIs by fluoroquinolones.

**Table 1:** Antibiotic resistance pattern of studied *E. coli *strains

**Antibiotic**	**Resistant**		**Intermediate- resistant**		**Susceptible**	
	No.	%	No.	%	No.	%
**Nalidixic acid**	138	61.9	7	3.1	78	35
**Ciprofloxacin**	112	50.2	7	3.1	104	46.6
**Ofloxacin**	109	48.9	2	0.9	112	50.2
**Norfloxacin**	101	45.3	1	0.4	121	54.3

**Table 2:** Minimum inhibitory concentrations (MICs) and susceptibility profiles of *E. coli *isolates toward the tested antimicrobial agents

**Antibiotics**	**MIC value of strip**	**MIC50 (µg/mL)**	**MIC90 (µg/mL)**	**MIC range (µg/mL)**	**Susceptible rate (%)**
**Nalidixic acid**	0.016-256	32	>256	2-256	35
**Ciprofloxacin**	0.002-32	16	>32	0.094-32	46.6
**Ofloxacin**	0.002-32	4	>32	0.094-32	50.2
**Norfloxacin**	0.016-256	8	128	0.38-256	54.3

**Table 3:** Relation between mutation in codons 83 and 106 *gyrA *gene and ciprofloxacin resistance among *E. coli *isolates

**Resistance pattern**		**Resistant**		**Intermediate- resistant**		**Susceptible**		***P*** **-value**
**Mutation**		No.	%	No.	%	No.	%	
***gyrA*** ** 83**	**Yes**	73	59.3	1	0.8	49	39.8	0.002
	**No**	39	39	6	6	55	55	0.002
***gyrA*** ** 106**	**Yes**	41	91.1	0	0	4	8.9	<0.001
	**No**	71	39.9	7	3.9	100	56.2	<0.001
***gyrA *** **83 &106**	**Yes**	30	88.2	0	0	4	11.8	<0.001
	**No**	82	43.4	7	3.7	100	52.9	<0.001

**Table 4:** Association between mutation in codons 83 and 106 *gyrA *gene and norfloxacin resistance among *E. coli *isolates

**Resistance pattern**		**Resistant**		**Intermediate- resistant**		**Susceptible**		***P*** **-value**
**Mutation**		No.	%	No.	%	No.	%	
***gyrA*** ** 83**	**Yes**	68	55.3	1	0.8	53	43.9	0.002
	**No**	33	33	0	0	67	67	0.002
***gyrA*** ** 106**	**Yes**	38	84.4	0	0	7	15.6	<0.001
	**No**	63	35.4	1	0.6	114	64	<0.001
***gyrA *** **83 &106**	**Yes**	30	88.2	0	0	4	11.8	<0.001
	**No**	71	37.6	1	0.5	117	61.9	<0.001

**Table 5:** Association between mutation in codons 83 and 106 *gyrA *gene and resistance to ofloxacin among *E. coli *isolates

**Resistance pattern**		**Resistant**		**Intermediate- resistant**		**Susceptible**		***P*** **-value**
**Mutation**		No.	%	No.	%	No.	%	
***gyrA*** ** 83**	**Yes**	72	58.5	1	0.8	50	40.7	0.006
	**No**	37	37	1	1	62	62	0.006
***gyrA*** ** 106**	**Yes**	42	93.3	0	0	3	6.7	<0.001
	**No**	67	37.6	2	1.1	109	61.2	<0.001
***gyrA *** **83 &106**	**Yes**	31	91.2	0	0	3	8.8	<0.001
	**No**	78	41.3	2	1.1	109	57.7	<0.001

**Table 6:** Association between mutation in codons 83 and 106 *gyrA *gene and resistance to Nalidixic acid among *E. coli *isolates

**Resistance pattern**		**Resistant**		**Intermediate- resistant**		**Susceptible**		***P*** **-value**
**Mutation**		No.	%	No.	%	No.	%	
***gyrA*** ** 83**	**Yes**	93	75.6	3	2.4	27	22	<0.001
	**No**	45	45	4	4	51	51	<0.001
***gyrA*** ** 106**	**Yes**	39	86.7	1	2.2	5	11.1	0.001
	**No**	99	56.6	6	3.4	73	41	0.001
***gyrA *** **83 &106**	**Yes**	31	91.2	0	0	3	8.8	<0.001
	**No**	107	56.6	7	3.7	75	39.7	<0.001

In this study, we investigated the prevalence of mutations in the *gyrA* genes as one of the principal mechanism of fluoroquinolones resistance in Gram-negative bacteria. Our results showed the highest prevalence of mutations regarded to the *gyrA* gene in codon 83 with 55.2% followed by mutation in codon 106 with 20.2%. Previously, several studies from Iran and other countries showed alteration in the GyrA protein is mostly associated with quinolones resistance in *Enterobacteriaceae* and nonfermenting Gram-negative bacilli (4, 33-39).

Moreover, a double concomitant mutation in *gyrA* (codons 83 and 106) was observed in 15.2% of isolates. Previously, it has been documented that low-level fluoroquinolone resistance in *E. coli* is associated with a single mutation, while high-level resistance required double mutations (36). In accordance with this finding, we found the majority of double mutation (more than 80%) is associated with quinolone-resistant isolates.

Finally, despite the significant role of mutations in the QRDRs of *gyrA*, we found quinolone-resistant isolates without any mutations in this region. Therefore it is possible that other resistance mechanisms such as mutations in *parC* or the presence of horizontally acquired genes (*qnr* genes) may cause of quinolone resistance in our isolates (25, 36-38, 40).

As the main limitations of the present work, the lack of sequencing for confirming the RFLP results to reveals the exact status of base replacements among the mutant strains must be mentioned.

In summary, our results revealed a high level of quinolone resistance is associated with the mutations in *gyrA* among the clinical isolates of UPEC in our region. To the best of our knowledge, this study is the first investigation on the role of *gyrA* alteration in quinolone resistance among UPEC isolates from north of Iran.
